# Goitre Treated Successfully with Strophanthus

**Published:** 1889-09

**Authors:** S. T. Yount


### GOITRE TREATED SUCCESSFULLY WITH STROPHANTHUS.

By S. T. YOUNT, M. D.

Up to the present date I have treated successfully five cases of goitre without a single failure.

Miss Anna C———, aged 22 called on me Dec. 10th, 1888, suffering from "big neck," as she called it. Various remedies (such as ergot, bromides, and digitalis were given internally, and injections of carbolic acid and ergotine were made into the gland) were tried without any appreciable results. At last I prescribed strophanthus for her in ten drop doses three times a day. At the time she commenced taking strophanthus her neck measured fourteen inches. In ten days it measured thirteen inches, and in three weeks twelve inches. The strophanthus was given in ten drop doses three times a day for one week and then increased to twelve drops three times a day and finally up to sixteen drops three times a day. The enlargement subsided very rapidly and in two months she declared herself well and to all appearances she was cured. The only unpleasant features about the treatment in all cases that I have treated is the profound dizziness and faintness.

Miss Jennie R———, aged 16, called on me Jan. 8th, 1889, with an immense goitre. It measured thirteen and one-half inches. She was put upon the tincture of strophanthus and her recovery was as prompt and satisfactory as the first case reported. She was discharged cured, March 15th.

The other cases were similar to those reported and the recovery equally rapid.

My attention was first attracted to the value of strophanthus in goitre in a most singular manner. Last December Mrs. R———, sent for me to treat her for some heart trouble. She was short of breath, suffered from palpitation and had a very bad capillary circulation. She informed me that digitalis acted like a poison to her. She also showed me her goitre, an enormous one, and said that her former physician had given her ergot and digitalis for it, without any effect, save to make her deathly sick. She needed a heart tonic and I prescribed it for her in big doses (10 drops every four hours). I left her and saw no more of her for three weeks or more. When she did show up she was much improved, and the most astonishing part of all her neck was decidedly smaller. Her breathing was good and she felt much better, and she was greatly relieved, but never cured, for the simple reason she would not take it any longer.—*Med. Waif.*

